# Association between endocrine disrupting chemicals and female infertility: a study based on NHANES database

**DOI:** 10.3389/fpubh.2025.1608861

**Published:** 2025-06-30

**Authors:** Luo Bingru, Chen Ting, Zhang Zhe, Jiang Wen, Zeng Qianling, Zhu Hailun

**Affiliations:** ^1^Reproductive Medicine Center, The First-affiliated Hospital of Hunan Normal University (Hunan Provincial People's Hospital), Changsha, Hunan, China; ^2^Department of Obstetrics, The First-affiliated Hospital of Hunan Normal University (Hunan Provincial People's Hospital), Changsha, Hunan, China

**Keywords:** endocrine disrupting chemicals, PAEs, equol, PFASs, female infertility, NHANES

## Abstract

**Background:**

Controversy persists regarding the impact of endocrine disrupting chemicals (EDCs) on female infertility, and the specific EDCs that cause female infertility remain unclear. This study aims to examine the associations between various EDCs metabolites and female infertility using data from the female population in the National Health and Nutrition Examination Survey (NHANES) conducted between 2001 and 2006.

**Methods:**

A cross-sectional study on reproductive-age women aged 18–45 years was conducted, with data retrieved from the NHANES database. Multivariate logistic regression analysis was performed to evaluate the association between EDCs metabolites and female infertility. Subgroup analysis was applied to stratify by age and body mass index (BMI). Results were summarized using an odds ratio (OR) with a 95% confidence interval (CI).

**Results:**

A total of 3,982 women were enrolled, comprising 463 infertile women and 3,519 control women. The results showed that increased exposure to EDCs metabolites (including DnBP, DEHP, DiNP, DEHTP, PAEs, Equol, PFOA, and PFUA) was significantly associated with female infertility, with odds ratios of 2.10 (95% CI: 1.59, 2.48), 1.36 (95% CI: 1.05, 1.79), 1.62 (CI: 1.31, 1.97), 1.43 (95% CI: 1.22, 1.78), 1.43 (95% CI: 1.26, 1.75), 1.41 (95% CI: 1.17, 2.35), 1.34 (95% CI: 1.15, 2.67), and 1.58 (95% CI: 1.08, 2.03), respectively. Sensitivity analyses confirmed the robustness of these findings. The subgroup analysis also indicated that increased age and BMI may exacerbate the risk of female infertility among those exposed to EDCs metabolites.

**Conclusions:**

This study indicates that exposure to EDCs metabolites such as PAEs, equol, and PFASs are associated with female infertility. These findings provide valuable evidence for preventing female infertility from the perspective of EDCs exposure.

## Introduction

In addition to genetic and psychosocial factors, female infertility is influenced by external factors such as exposure to harmful substances in the environment. Endocrine disrupting chemicals (EDCs) constitute a group of exogenous chemicals or mixtures that enter the human body through environmental exposure or dietary intake. EDCs have liver toxicity. Studies have shown that PFAS can induce hepatic steatosis by activating the PPARα/γ pathway and inhibit mitochondrial β-oxidation (1). Female reproduction is regulated by hormones and is susceptible to the effects of exposure to EDCs. Katz et al. found that when EDC exposure occurs at the critical stage of uterine development, changes in cellular signaling pathways (including estrogen signaling) and epigenomes may increase the susceptibility to adult onset of uterine fibroids (2). Chiang et al. have confirmed through mice experiments that EDCs exposure have long-term effects on female reproduction, even after stopping exposure for a long time. Grindler et al. (3) found EDC-exposed women were up to six times more likely to be menopausal than non-exposed women. However, whether there is a relationship between EDCs exposure and a female infertility has not been explored on a large scale. The specific EDCs that cause female infertility remain unclear. This study aims to examine the associations between various EDCs metabolites (primarily phthalates, equol, per- and poly-fluoroalkyl substances) and female infertility using data from the female population in the National Health and Nutrition Examination Survey (NHANES) database.

## Materials and methods

### Study design and population

**Figure 1:**
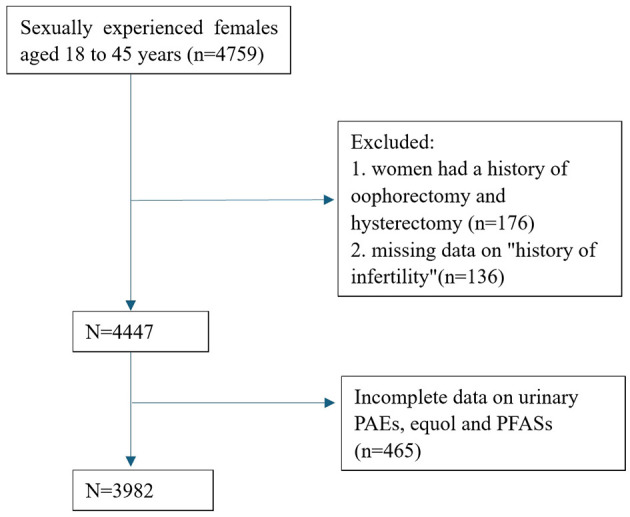
Flowchart of participant selection.

A cross-sectional study was conducted using data from the NHANES database spanning the years 2001 to 2006. NHANES is a research program designed to assess the health and nutritional status of the United States population since the 1960s. This survey comprises interviews and physical examinations of a nationally representative sample. NHANES collects information on demographics, socioeconomics, diet, and health-related factors. The physical examination component involves medical, dental, and physiological measurements, along with laboratory tests conducted under the guidance of qualified medical professionals (4). We selected data from sexually experienced females aged 18– 45 years. Women who did not respond to the question on “history of infertility”, or who had a history of oophorectomy and hysterectomy were excluded from the study. Female participants with incomplete measurements of urinary phthalate metabolites (PAEs), equol, and per- and poly-fluoroalkyl substances (PFASs) concentrations were excluded from further analysis. A flowchart depicting the data selection process is provided in Figure 1. Since the dataset included in this study was downloaded from a public database, approval from our hospital's ethics committee was not required. We adhered to the Strengthening the Reporting of Observational Studies in Epidemiology (STROBE) statement, guided by the Enhancing the QUAlity and Transparency Of health Research (EQUATOR) Network guidelines (5).

### EDCs

Following the standardized household interview conducted by the National Health and Nutrition Examination Survey (NHANES), a detailed physical examination is performed either at the mobile examination center (MEC) or within the participants' homes. Measurements included urinary PAEs, equol, and serum PFASs. Prior to being dispatched to the National Center for Environmental Health, Centers for Disease Control and Prevention (CDC) for processing, samples were collected at the MEC and stored at 30°C. We a priori limited our analysis to EDCs for which at least 60% of samples were > lower limit of detection (LLOD), thus we included 10 urinary PAEs, equol and seven serum PFAS. For analysis with results below the LLOD, imputation filling values were provided in the analyte result fields, calculated by dividing the LLOD by the square root of 2 (LLOD/√2).

### Self-reported infertility

Infertility is defined as the inability to conceive after one year of unprotected sexual intercourse (6). The presence of infertility was assessed using self-reported questionnaires.

### Covariate

The selection of covariates was based on previous studies on the association between EDCs and female infertility. A comprehensive set of demographic, socioeconomic, lifestyle and health-related variables were included to adjust for potential confounding effects. Through questionnaires, physical examinations, and laboratory tests, we obtained demographic variables on age, body mass index (BMI, kg/m^2^), race (non-Hispanic white, non-Hispanic black, Hispanic, or other). Socioeconomic factors included educational attainment (less than high school graduate, high school graduate or equivalent, above high school), household income (under 20,000 and above 20,000), marital status (married/cohabiting, widowed/divorced/separated, never married). Lifestyle factors included smoking status and alcohol use. The health-related variables included history of menstrual, pelvic infections, metabolic syndrome or virus hepatitis (infection with hepatitis B virus or hepatitis C virus).

### Statistical analysis

The *t*-test was used to compare group differences of continuous variables, which are shown as the mean and standard deviation (SD). Counting data were expressed as numbers and percentages were compared between the two groups using the chi-square test. Normality of variables was assessed using Shapiro–Wilk tests. For non-normally distributed variables, non-parametric tests (Mann–Whitney *U*) were applied. *T*-tests were used only for variables meeting normality assumptions. EDCs concentration was classified into quartiles (Q1: P0–25, Q2: P25–50, Q3: P50–75, Q4: P75–100). Logistic regression model was used to evaluate the association between log-transformed EDCs metabolites and female infertility, and the strength of the association was determined with the odds ratio (OR) and confidence interval (CI). Model 1 was created by only adjusted for creatinine, and Model 2 was adjusted for age, BMI, race, educational attainment, household income, marital status. Model 3, based on model 2, was further adjusted for menstrual history, smoking status, alcohol use and history of pelvic infections, metabolic syndrome or virus hepatitis.

Sensitivity analyses were performed to evaluate the impact of potential outliers, participants with EDCs concentrations above the 99th percentile were excluded. To eliminate the influence of taking the lowest measurable concentration when the EDCs concentration is lower than the measured value, the EDCs concentration below the measured value is randomly reassigned, and all analyses were rerun on the subset to compare with the main analysis results. The subgroup analysis was performed based on age and BMI. Statistical significance was defined as a *P* value of < 0.05. The data were analyzed using R software (R 3.6.0).

## Results

### Basic characteristics of included participants

In this study, a total of 3,982 women were enrolled, comprising 463 infertile women and 3,519 control women. Table 1 presents the baseline characteristics of the study population. The infertile group exhibited significantly higher ages and BMI compared to the control group (*P* < 0.05 for both comparisons). A statistically significant difference was observed in marital status between infertile women and control women (*P* < 0.05). No significant differences were found between the two groups in terms of ethnicity, educational level, annual family income, menstrual regularity, pelvic infections, smoking history, alcohol consumption, metabolic syndrome and virus hepatitis (all *P* > 0.05).

**Table 1:** Basic characteristics of included participants.

**Characteristics**	**Female infertility**		***P***
	**Yes (*****n*** = **463)**	**No (*****n*** = **3519)**	
Age year (SD)	29 ± 5.21	34 ± 6.72	<0.05
BMI	27.32	31.47	0.02
**Ethnicity %**			0.32
Non-Hispanic white	55.32	53.65	
Non-Hispanic black	16.76	17.81	
Hispanic/other	27.92	28.54	
**Education level %**			0.64
Less than high school graduate	23.12	21.31	
High school graduate or equivalent	23.64	21.97	
Above high school	53.24	56.72	
**Marital status %**			<0.05
Marry/Conhabity	41.52	63.78	
Widowed/Divorced/Separated	22.63	25.14	
Never married	35.85	11.08	
**Annual family income %**			0.73
$20,000 over	79.32	81.65	
Under $20,000	20.68	18.35	
**Regular menstrual periods %**			0.13
Yes	85.32	89.15	
No	14.68	10.85	
**Pelvic infection %**			0.16
Yes	9.68	6.55	
No	90.32	93.45	
**Smoking history %**			0.25
Yes	13.51	17.32	
No	86.49	82.68	
**Alcohol use %**			0.26
Yes	54.32	60.54	
No	45.68	39.46	
**Metabolic syndrome %**			0.42
Yes	25.13	22.64	
No	74.87	77.35	
**Virus hepatitis %**			0.53
Yes	1.30	1.42	
No	98.70	98.58	

The distribution of EDCs metabolites are presented in Supplementary Table 1. The detection rates of PAEs in urine samples ranged from 57.31% to 98.96%, with equol detected in 57.43% of the samples. In blood samples, the detection rates of PFASs were between 43.28% and 99.45%. Di-2-ethylhexyl terephthalate (DEHTP) exhibited the highest mean concentration, followed by Di-iso-nonyl phthalate (DiNP) and Di-n-butyl phthalate (DnBP), with mean concentrations of 169.41 ng/ml, 135.32 ng/ml, and 32.15 ng/ml, respectively. The concentration of equol was 78.65 ng/ml. Among the PFASs tested, perfluorooctanoic acid (PFOA) had the highest mean concentration, averaging at 12.56 μg/l.

### Association between EDCs and female infertility

Table 2 summarizes the associations between exposure to EDCs metabolites and female infertility. We found that exposure to EDCs metabolites was significantly associated with female infertility. PAEs demonstrated the strongest correlation with female infertility, with odds ratios (ORs) ranging from 0.75 to 2.10. For instance, in Model 3, the DnBP (OR: 2.10, 95% CI: 1.59, 2.48), DEHP (OR: 1.36, 95% CI: 1.05, 1.79), DiNP (OR: 1.62, 95% CI: 1.31, 1.97), DEHTP (OR: 1.43, 95% CI: 1.22, 1.78) and PAEs (OR: 1.43, 95% CI: 1.26, 1.75) were positively associated with female infertility. Compared to the first quartile (Q1), the OR of the Q4 for PAEs on female infertility was 1.46 (95% CI: 1.23, 1.54).

**Table 2:** Odds ratio (95% confidence interval) of female infertility associated with EDCs.

		**Model 1 (OR 95%CI)**	***P***	**Model 2 (OR 95%CI)**	***P***	**Model 3 (OR 95%CI)**	***P***
DEP (ng/mL)	Q1	Ref.	0.14	Ref.	0.51	Ref.	0.06
	Q2	1.54 (0.76, 1.89)		1.62 (0.86, 1.85)		1.29 (0.71, 1.62)	
	Q3	1.35 (0.82, 1.74)		1.45 (0.76, 1.78)		**1.09 (1.02, 1.33)**	
	Q4	1.08 (0.35, 1.23)		**1.23 (1.05, 1.65)**		**1.14 (1.09.1.47)**	
	Log10	**1.12 (1.03, 1.82)**		1.19 (0.78, 1.57)		1.18 (0.86, 1.44)	
DiBP (ng/mL)	Q1	Ref.	0.39	Ref.	0.08	Ref.	0.61
	Q2	1.05 (0.63, 1.41)		0.84 (0.57, 1.24)		0.95 (0.52, 1.56)	
	Q3	1.06 (0.79, 1.46)		0.83 (0.56, 1.19)		0.83 (0.36, 1.66)	
	Q4	0.85 (0.62, 1.21)		0.68 (0.45, 0.98)		0.78 (0.45, 1.55)	
	Log10	0.87 (0.66, 1.23)		0.72 (0.51, 1.03)		0.75 (0.52, 1.64)	
DnBP (ng/mL)	Q1	Ref.	**0.00**	Ref.	**0.00**	Ref.	**0.00**
	Q2	1.06 (0.75, 1.41)		0.76 (0.65, 1.41)		**1.15 (1.12, 1.84)**	
	Q3	**1.12 (1.06, 1.54)**		**1.32 (1.13, 1.51)**		**1.24 (1.16, 2.36)**	
	Q4	**1.67 (1.43, 1.98)**		**1.70 (1.51, 1.92)**		**1.92 (1.50, 2.54)**	
	Log10	**1.82 (1.67, 2.12)**		**1.83 (1.45, 1.95)**		**2.10 (1.59, 2.48)**	
BBzP (ng/mL)	Q1	Ref.	0.41	Ref.	0.33	Ref.	0.75
	Q2	1.34 (0.91, 1.72)		0.95 (0.73, 1.54)		1.23 (0.65, 2.47)	
	Q3	1.02 (0.81, 1.53)		0.92 (0.72, 1.42)		1.15 (0.32, 2.16)	
	Q4	0.93 (0.72, 1.32)		0.78 (0.54, 1.07)		1.35 (0.63, 2.37)	
	Log10	0.94 (0.76, 1.16)		0.81 (0.62, 1.08)		1.25 (0.66, 1.89)	
DEHP (ng/mL)	Q1	Ref.	0.32	Ref.	0.42	Ref.	**0.00**
	Q2	0.86 (0.45, 1.32)		1.07 (0.65, 1.57)		1.13 (0.76, 1.35)	
	Q3	1.35 (0.78, 1.45)		1.24 (0.81.1.63)		**1.34 (1.12, 1.53)**	
	Q4	**1.52 (1.31, 1.85)**		**1.35 (1.14, 1.77)**		**1.23 (1.18, 1.64)**	
	Log10	**1.61 (1.12, 1.93)**		1.47 (0.39, 1.82)		**1.36 (1.05, 1.79)**	
DnOP (ng/mL)	Q1	Ref.	0.35	Ref.	0.79	Ref.	0.14
	Q2	0.85 (0.62, 1.19)		0.74 (0.57, 1.16)		0.95 (0.54, 1.57)	
	Q3	1.18 (0.74, 1.63)		0.83 (0.64, 1.25)		1.47 (0.85, 2.56)	
	Q4	1.26 (0.95, 1.63)		0.95 (0.62, 1.48)		1.43 (0.76, 2.73)	
	Log10	1.35 (0.96, 1.72)		1.02 (0.64, 1.76)		1.35 (0.86, 2.47)	
DiNP (ng/mL)	Q1	Ref.	0.32	Ref.	**0.02**	Ref.	**0.00**
	Q2	0.56 (0.34, 1.12)		1.03 (0.68, 1.38)		**1.22 (1.07, 1.56)**	
	Q3	0.86 (0.52, 1.35)		**1.28 (1.06, 1.45)**		**1.34 (1.15, 1.68)**	
	Q4	**1.24 (1.06, 1.32)**		**1.14 (1.05, 1.37)**		**1.53 (1.22, 1.86)**	
	Log10	**1.38 (1.16, 1.59)**		**1.30 (1.15.1.53)**		**1.62 (1.31, 1.97)**	
DiDP (ng/mL)	Q1	Ref.	0.40	Ref.	0.34	Ref.	0.35
	Q2	1.02 (0.87, 1.34)		0.83 (0.65, 1.35)		1.24 (0.75, 1.57)	
	Q3	1.34 (0.92, 1.65)		1.05 (0.87, 1.42)		1.31 (0.83, 1.48)	
	Q4	1.42 (0.96, 1.75)		1.21 (0.78, 1.65)		1.64 (0.91, 1.56)	
	Log10	1.55 (0.87.1.88)		1.53 (0.67, 1.84)		1.75 (0.97, 1.86)	
DEHTP (ng/mL)	Q1	Ref.	**0.00**	Ref.	**0.00**	Ref.	**0.00**
	Q2	1.31 (0.78, 1.54)		**1.37 (1.02, 1.62)**		**1.23 (1.16, 1.59)**	
	Q3	**1.42 (1.06, 1.68)**		**1.56 (1.22, 1.76)**		**1.35 (1.06, 1.62)**	
	Q4	**1.35 (1.16, 1.82)**		**1.12 (1.09, 1.35)**		**1.37 (1.15, 1.73)**	
	Log10	**1.45 (1.21, 1.76)**		**1.21 (1.05, 1.42)**		**1.43 (1.22, 1.78)**	
DINCH (ng/mL)	Q1	Ref.	0.56	Ref.	0.72	Ref.	0.51
	Q2	0.97 (0.64, 1.42)		0.88 (0.67, 1.412)		1.28 (0.56, 1.73)	
	Q3	0.96 (0.75, 1.28)		1.08 (0.77, 1.45)		1.14 (0.75, 1.86)	
	Q4	1.01 (0.83, 1.43)		1.12 (0.82, 1.54)		1.15 (0.67, 1.76)	
	Log10	1.16 (0.96, 1.39)		1.36 (0.99, 1.63)		1.24 (0.85, 1.94)	
PAEs (ng/mL)	Q1	Ref.	0.64	Ref.	0.31	Ref.	**0.02**
	Q2	1.06 (0.76, 1.45)		1.14 (0.76, 1.56)		1.17 (0.85, 1.42)	
	Q3	1.15 (0.86, 1.76)		1.22 (0.72, 1.65)		**1.21 (1.07, 1.55)**	
	Q4	1.32 (0.88, 1.75)		1.26 (0.67, 1.78)		**1.46 (1.23, 1.54)**	
	Log10	1.42 (0.76, 1.82)		1.36 (0.75, 1.57)		**1.43 (1.26, 1.75)**	
Equol (ng/mL)	Q1	Ref.	0.45	Ref.	0.32	Ref.	**0.04**
	Q2	1.32 (0.87, 1.45)		0.95 (0.68, 1.43)		1.16 (0.65, 1.42)	
	Q3	1.42 (0.91, 1.56)		1.32 (0.76, 1.57)		**1.23 (1.05, 1.56)**	
	Q4	1.45 (0.92, 1.58)		1.29 (0.67, 1.74)		**1.32 (1.13, 1.67)**	
	Log10	1.52 (0.93, 1.62)		1.43 (0.72, 1.78)		**1.41 (1.17, 2.35)**	
PFOA (ng/mL)	Q1	Ref.	**0.00**	Ref.	0.403	Ref.	**0.00**
	Q2	**1.55 (1.16, 2.04)**		1.28 (0.84, 1.73)		1.42 (0.65, 2.43)	
	Q3	**1.52 (1.05, 1.98)**		1.12 (0.74, 1.64)		**1.44 (1.26, 2.43)**	
	Q4	**1.27 (1.02, 1.63)**		**1.27 (1.14, 1.95)**		**1.58 (1.39, 2.85)**	
	Log10	**1.13 (1.08, 1.45)**		1.12 (0.74, 1.65)		**1.34 (1.15, 2.67)**	
PFOS (ng/mL)	Q1	Ref.	0.41	Ref.	0.51	Ref.	0.54
	Q2	0.86 (0.32, 1.65)		0.85 (0.66, 2.65)		0.65 (0.83, 2.94)	
	Q3	1.43 (0.56, 1.54)		1.23 (0.82, 1.53)		1.13 (0.64, 1.68)	
	Q4	1.87 (0.98, 1.47)		1.45 (0.65, 1.45)		1.26 (0.64, 1.93)	
	Log10	1.56 (0.45, 1.67)		1.34 (0.82, 1.78)		1.20 (0.46, 1.74)	
PFDeA (ng/mL)	Q1	Ref.	0.67	Ref.	0.23	Ref.	0.71
	Q2	1.12 (0.65.1.42)		1.24 (0.75, 1.62)		1.25 (0.73, 1.78)	
	Q3	1.23 (0.75, 1.45)		1.25 (0.67, 1.72)		1.37 (0.82, 1.67)	
	Q4	1.26 (0.73, 1.54)		**1.36 (1.07, 1.76)**		1.41 (0.83, 1.57)	
	Log10	1.34 (0.85, 1.63)		1.44 (0.57, 1.71)		1.53 (0.71, 1.68)	
PFHxS (ng/mL)	Q1	Ref.	0.21	Ref.	0.21	Ref.	0.51
	Q2	0.75 (0.67, 1.23)		0.83 (0.42, 1.35)		0.48 (0.32, 1.69)	
	Q3	1.35 (0.74, 2.21)		1.43 (0.76, 1.98)		1.65 (0.84, 2.62)	
	Q4	2.11 (0.96, 2.65)		1.53 (0.78, 2.34)		1.64 (0.66, 1.98)	
	Log10	1.32 (0.65, 1.94)		1.76 (0.87, 2.35)		1.83 (0.77, 2.19)	
PFOSA (ng/mL)	Q1	Ref.	0.32	Ref.	0.76	Ref.	0.64
	Q2	0.78 (0.65, 1.32)		1.05 (0.87, 1, 66)		1.01 (0.65, 1.36)	
	Q3	1.12 (0.57, 1.33)		1.07 (0.91, 1.68)		1.08 (0.75, 1.32)	
	Q4	1.24 (0.78, 1.42)		1.13 (0.87, 1.72)		1.18 (0.91, 1.47)	
	Log10	1.32 (0.89, 1.56)		1.33 (0.96, 1.54)		1.12 (0.85, 1.39)	
PFNA (ng/mL)	Q1	Ref.	0.72	Ref.	0.65	Ref.	0.59
	Q2	1.05 (0.67, 1.42)		1.06 (0.78, 1.34)		1.12 (0.89, 1.45)	
	Q3	1.09 (0.83, 1.65)		1.22 (0.91, 1.45)		1.27 (0.63, 1.59)	
	Q4	1.21 (0.87, 1.32)		1.20 (0.85, 1.62)		1.28 (0.86, 1.78)	
	Log10	1.22 (0.69, 1.43)		1.67 (0.68, 1.79)		1.48 (0.45, 1.58)	
PFUA (ng/mL)	Q1	Ref.	**0.00**	Ref.	0.481	Ref.	**0.03**
	Q2	**0.76 (0.53, 0.91)**		0.65 (0.45, 0.94)		0.86 (0.58, 1.44)	
	Q3	**0.73 (0.74, 0.96)**		0.83 (0.65, 1.34)		1.07 (0.65, 1.62)	
	Q4	**0.45 (0.42, 0.46)**		0.82 (0.56, 1.24)		**1.35 (1.02, 2.15)**	
	Log10	**0.67 (0.34, 0.67)**		0.78 (0.53, 1.43)		**1.58 (1.08, 2.03)**	
PFASs (ng/mL)	Q1	Ref.	0.21	Ref.	0.32	Ref.	0.43
	Q2	0.92 (0.65, 1.35)		0.81 (0.45, 1.76)		1.26 (0.78, 2.13)	
	Q3	1.56 (0.76, 2.13)		1.14 (0.65, 1.82)		1.32 (0.85, 1.94)	
	Q4	1.92 (0.87, 2.31)		1.46 (0.72, 1.79)		1.42 (0.58, 1.76)	
	Log10	2.04 (0.78, 2.45)		1.89 (0.85, 2.45)		2.34 (0.68, 2.75)	

All results of endocrine–disrupting chemicals metabolites had been log–transformed. Model 1: only adjusted for creatinine. Model 2 adjusted for age, BMI, race, educational attainment, household income, marital status. Model 3 based on model 2 and further adjusted for menstrual history, smoking status, alcohol use and history of pelvic infections, metabolic syndrome or virus hepatitis. This analysis was carried out in a logistic regression model. Log10 represented the metabolite concentration had been log–transformed for analysis. The statistically significant indices were marked in bold (*P* < 0.05).

A statistically significant association was found between equol and female infertility. In Model 3, log-transformed equol demonstrated a positive correlation with female infertility (OR: 1.41, 95% CI: 1.17, 2.35). There was a greater reported OR for female infertility in the Q4 group of log-transformed equol metabolites than in the Q1 group (OR: 1.32, 95% CI: 1.13, 1.67).

PFASs were also significantly associated with female infertility. For example, in Model 3, log-transformed PFOA (OR: 1.34, 95% CI: 1.15, 2.67) and perfluoroundecanoic acid (PFUA) (OR: 1.58, 95% CI: 1.08, 2.03) were positively associated with female infertility. Logistic regression analysis revealed that log-transformed PFOA (OR: 1.58, 95% CI: 1.39, 2.85) and PFUA (OR: 1.35, 95% CI: 1.02, 2.15) exposure were positively associated with female infertility in the Q4 group.

### Sensitivity analysis

To evaluate the impact of potential outliers, we conducted sensitivity analyses by excluding participants whose EDCs concentrations were above the 99th percentile (Supplementary Table 2). The results remained largely consistent with our main analysis. To evaluate the impact of EDCs at its lowest measurable concentration, we conducted sensitivity analyses by randomly reassigned EDCs to the lowest measurable concentration (Supplementary Table 3). In sensitivity analysis, the OR and the *P*-value in Model 1 was consistent with our main analysis results, indicating a significant correlation between EDCs and female infertility. All EDCs *P*-values in Model 2 and Model 3 were consistent with our main analysis results. Therefore, results of the sensitivity analysis demonstrated that the conclusions drawn in the present study are stable and reliable.

### Stratification analysis

Age and BMI were both significant factors contributing to female infertility. In this study, stratified analyses were conducted based on these two factors. Logistic regression was utilized to assess the relationship between EDCs metabolites and infertility, with adjustments for race, education level, marital status, annual family income, regular menstrual cycles, smoking history, alcohol use and history of pelvic infections, metabolic syndrome or virus hepatitis.

Our results indicate that the correlation between EDCs metabolites exposure and female infertility varies with age (Table 3). Among infertile women aged ≥35, DEP (OR: 4.76, 95% CI 1.63,10.75), Equol (OR: 6.47, 95% CI 1.64, 9.32), PFOS (OR: 3.34, 95% CI 1.57,9.63) and perfluorohexane sulfonic acid (PFHxS) (OR: 6.45, 95% CI 1.73, 10.32) were positively associated with infertility. In individuals aged <35, PFOA (OR: 1.53, 95% CI 1.24, 4.26) was positively associated with infertility.

**Table 3:** Stratified analyses based on age and BMI.

	**Age**	**OR (95% CI)**	***P***		**BMI**	**OR (95% CI)**	***P***
DEP	<35	1.62 (0.63, 6.74)		DEP	<24	2.03 (0.92, 4.53)	
	≥35	**4.76 (1.63, 10.75)**	0.12		≥24	**1.83 (1.11, 2.43)**	0.23
DiBP	<35	1.35 (0.43, 6.35)		DiBP	<24	1.32 (0.51, 6.04)	
	≥35	0.96 (0.17, 4.32)	0.55		≥24	1.78 (0.43, 2.86)	0.32
DnBP	<35	2.34 (0.86, 6.37)		DnBP	<24	1.58 (0.39, 3.67)	
	≥35	0.95 (0.41, 3.62)	0.75		≥24	0.93(0.62, 1.72)	0.75
BBzP	<35	1.15 (0.67, 4.32)		BBzP	<24	0.87(0.45, 1.57)	
	≥35	0.96 (0.65, 1.78)	0.68		≥24	0.93 (0.67, 1.37)	0.75
DEHP	<35	1.78 (0.58, 6.78)		DEHP	<24	0.59 (0.15, 1.22)	
	≥35	0.52 (0.36, 3.45)	0.71		≥24	1.17 (0.75, 3.86)	0.25
DnOP	<35	1.68 (0.65, 5.25)		DnOP	<24	0.92 (0.67, 2.79)	
	≥35	0.74 (0.32, 3.65)	0.67		≥24	1.56 (0.67, 2.46)	0.73
DiNP	<35	1.43 (0.66, 3.62)		DiNP	<24	1.57 (0.54, 2.45)	
	≥35	0.74 (0.55, 3.57)	0.21		≥24	1.16 (0.84, 2.97)	0.41
DiDP	<35	1.43 (0.25, 6.71)		DiDP	<24	1.73 (0.57, 6.82)	
	≥35	2.83 (0.32, 5.64)	0.85		≥24	1.23 (0.76, 4.65)	0.63
DEHTP	<35	1.45 (0.72, 6.47)		DEHTP	<24	1.34 (0.44, 2.87)	
	≥35	1.65 (0.87, 4.21)	0.51		≥24	1.65 (0.54, 3.58)	0.84
DINCH	<35	2.34 (0.96, 6.48)		DINCH	<24	1.73 (0.85, 5.27)	
	≥35	2.36 (0.89, 7.46)	0.75		≥24	1.93 (0.95, 6.61)	0.53
PAEs	<35	1.63 (0.86, 3.26)		PAEs	<24	1.68 (0.61, 5.83)	
	≥35	1.23 (0.64, 4.68)	0.63		≥24	1.35 (0.47, 4.21)	0.26
Equol	<35	1.32 (0.86, 3.78)		Equol	<24	1.68 (0.83, 4.37)	
	≥35	**6.47 (1.64, 9.32)**	**0.03**		≥24	**1.62 (1.13, 3.46)**	0.25
PFOA	<35	**1.53 (1.24, 4.26)**		PFOA	<24	1.12 (0.54, 3.62)	
	≥35	3.45 (0.76, 6.84)	0.57		≥24	**1.23 (1.06, 3.67)**	0.53
PFOS	<35	1.42 (0.75, 6.42)		PFOS	<24	1.27 (0.72, 4.57)	
	≥35	**3.34 (1.57, 9.63)**	0.18		≥24	1.44 (0.56, 3.42)	0.37
PFDeA	<35	0.93 (0.35, 2.62)		PFDeA	<24	1.25 (0.35, 3.78)	
	≥35	0.64 (0.16, 4.34)	0.81		≥24	1.52 (0.61, 4.53)	0.46
PFHxS	<35	**6.45 (1.73, 10.32)**		PFHxS	<24	1.36 (0.68, 3.21)	
	≥35	4.51 (0.23, 2.47)	0.31		≥24	1.54 (0.72, 3.16)	0.81
PFNA	<35	2.86 (0.65, 6.15)		PFNA	<24	1.63 (0.85, 4.34)	
	≥35	1.43 (0.74, 6.36)	0.82		≥24	1.26 (0.81, 4.54)	0.63
PFUA	<35	1.56 (0.53, 5.68)		PFUA	<24	2.37 (0.75, 5.37)	
	≥35	1.23(0.34, 5.36)	0.55		≥24	1.27 (0.62, 3.46)	0.23
PFASs	<35	1.25 (0.42, 4.38)		PFASs	<24	1.85 (0.53, 6.31)	
	≥35	1.13 (0.26, 5.37)	0.43		≥24	2.35 (0.43, 5.25)	0.56

The statistically significant indices were marked in bold (*P* < 0.05).

The correlation between EDCs metabolites exposure and female infertility also changes with BMI values (Table 3). Among infertile women with BMI ≥24, DEP (OR: 1.83, 95% CI 1.11, 2.43), Equol (OR: 1.62, 95% CI 1.13, 3.46), and PFOA (OR: 1.23, 95% CI 1.06, 3.67) were positively associated with infertility.

## Discussion

Female reproduction is a complex process regulated by various hormones and susceptible to the influence of EDCs. The impact of EDCs on female reproductive function may lead to reduced fertility, infertility, abnormal hormone production and irregular menstrual cycles. Recent studies have confirmed the potential effects of exposure to EDCs on the female reproductive system (7–10). Lin et al. found an association between exposure to heavy metal ions, which are EDCs, and urinary arsenic (As) was significantly related to female infertility, with a higher urinary As level indicating a greater risk of infertility (11). There was also a correlation between urinary cadmium (Cd) and infertility. Zhang et al. (12) found a positive relationship between equol and Hg exposure and uterine leiomyomata. However, the relationship between other EDCs (such as PAEs, equol, and PFASs) and female infertility remains unclear. This study analyzed the association between EDCs and female infertility based on data from the National Health and Nutrition Examination Survey (NHANES) 2001–2006. Our results indicated that exposure to EDCs metabolites such as PAEs, equol, and PFASs are associated with female infertility. Additionally, increased age and BMI may exacerbate the risk of female infertility among those exposed to EDCs.

PAEs are plasticizers used to impart flexibility to plastic products. PAEs may exert effects on the female reproductive system (13–15). For instance, DEHP, one of the most common PAEs, can leach out from products and cause toxic effects (16). PAEs have short half-lives and rapidly hydrolyze into biologically active monoesters and secondary metabolites upon ingestion, which are primarily excreted through urine and feces. Although PAEs have low bioaccumulation potential, their ubiquitous presence in the environment results in continuous exposure throughout different life stages. This sustained exposure allows PAEs to interact with other chemicals, potentially leading to synergistic, additive, or antagonistic effects, playing significant toxic roles in human reproductive development. A study in Israel examined 17 PAEs in the urine of 136 patients undergoing IVF due to male factor or unexplained infertility and found a negative correlation between the concentrations of DEHP metabolites in urine and the total number of oocytes retrieved, mature oocytes, fertilized oocytes, and high-quality embryos (17). However, recent research has indicated that the concentrations of PAEs in follicular fluid are not associated with clinical pregnancy rates and live birth rates following fresh and frozen-thawed embryo transfers (18). Our study shows a positive correlation between DnBP, DEHP, DiNP and DEHTP with female infertility.

Equol is a non-steroidal flavonoid estrogen that is a final metabolite produced from the metabolism of soy isoflavones by specific intestinal bacteria in the body. Equol exhibits potent estrogenic activity. Studies have reported an association between equol and uterine leiomyomas in women (19). An animal study found that equol had no significant effect on the ovaries but could induce uterine tissue hyperplasia by increasing epithelial cell height and the thickness of the uterine myometrium and stroma. This effect is long-term and may further contribute to the development of uterine leiomyomas (20). Further assessment of the potential health risks associated with soy food consumption in women of reproductive age is warranted. Our study demonstrates a positive correlation between equol and female infertility.

PFASs are synthetically produced chemical surfactants that are widely used in numerous industries and daily life applications. Due to their stable structure and resistance to degradation, PFASs can not only persist widely in contaminated environments but also enter the human body through dietary intake, drinking water, and respiration, accumulating through bioaccumulation and inducing adverse biological effects (21). A study by Dominguez et al. suggested that PFOS may impair oocyte maturation by disrupting gap junctions between oocytes and granulosa cells during the initial stages of cumulus-oocyte complex formation, thereby affecting clinical outcomes (22). A case-control study from the United States indicated a correlation between PFASs exposure and polycystic ovary syndrome (PCOS). Vagi et al. reported that the mean concentrations of PFOA and PFOS were significantly higher in the PCOS case group compared to the control group (23). However, another case-control study involving Chinese women showed no significant difference in blood PFOA levels between PCOS patients and the control group (24). These inconsistent results may be attributed to differences in menstrual characteristics and reproductive histories among participants. Our study demonstrates a positive correlation between PFOA exposure and female infertility.

This study is also subject to several limitations. Firstly, given its cross-sectional design, it is not possible to establish causal relationships between the studied variables. Secondly, the database lacks results for crucial confounding factors such as sexual frequency and menstrual cycle length, which may have influenced our findings. Thirdly, “infertility outcomes” are based on participants' self-reported answers rather than definitive infertility diagnoses, potentially introducing bias. Additionally, single PFAS measurement could not assess the impact of long-term exposure; Failure to include dietary data may lead to residual confounding; Cross section design cannot prove mutual reverse cause and effect. Beyond the EDCs examined here, other contaminants such as dioxins and PCBs may synergistically affect reproductive health. For instance, in Taranto, Italy, chronic dietary exposure to dioxins via contaminated animal products has been linked to altered estrogen signaling and ovulatory dysfunction (25). Although our study did not measure these compounds, future research should explore co-exposure scenarios, particularly in regions with industrial pollution to elucidate their associations with female infertility.

## Conclusions

In summary, this study indicates that exposure to EDCs such as PAEs, equol, and PFASs are associated with female infertility. Additionally, women with elevated BMI or advanced maternal age exhibited heightened susceptibility to EDC-related infertility. Suggesting that healthcare providers could integrate EDCs exposure assessments into preconception counseling for these groups, utilizing biomarkers to identify at-risk individuals. Policy interventions, such as stricter regulation of EDCs in consumer products and targeted biomonitoring programs, are warranted.

## Data Availability

The datasets presented in this study can be found in online repositories. The names of the repository/repositories and accession number(s) can be found in the article/supplementary material.
