# Supplementary material for: Association between endocrine disrupting chemicals and female infertility: a study based on NHANES database
Source: Front Public Health. 2025 Jun 30;13:1608861. doi: 10.3389/fpubh.2025.1608861 (PMC12257772; doi:10.3389/fpubh.2025.1608861)
Supplement: Supplementary file 1 [file Table_1.doc]

Table S1. Descriptive statistics of EDCs concentration and urine creatinine.

| Variables | Detection rates(%) | Mean | SD | Percentiles | | | | |
| --- | --- | --- | --- | --- | --- | --- | --- | --- |
|  |  |  |  | Min | 25th | 50th | 75th | Max |
| DEP (ng/mL) | 93.42 | 14.37 | 58.32 | 2.25 | 3.31 | 7.45 | 15.56 | 867.34 |
| DiBP (ng/mL) | 67.82 | 17.86 | 33.25 | 0.75 | 6.07 | 10.33 | 21.51 | 738.25 |
| DnBP (ng/mL) | 96.72 | 32.15 | 43.12 | 1.43 | 6.72 | 18.32 | 35.74 | 753.56 |
| BBzP (ng/mL) | 73.86 | 4.65 | 10.23 | 0.35 | 2.40 | 3.95 | 8.35 | 321.29 |
| DEHP (ng/mL) | 85.94 | 16.73 | 22.74 | 0.63 | 5.25 | 13.67 | 19.76 | 425.34 |
| DnOP (ng/mL) | 57.31 | 2.27 | 45.32 | 0.54 | 0.96 | 1.75 | 3.65 | 1320.87 |
| DiNP (ng/mL) | 97.64 | 135.32 | 451.45 | 0.76 | 10.56 | 35.64 | 75.64 | 6781.35 |
| DiDP (ng/mL) | 62.35 | 2.36 | 7.85 | 0.32 | 1.35 | 2.76 | 4.50 | 95.78 |
| DEHTP (ng/mL) | 98.96 | 169.41 | 1849.63 | 0.83 | 11.45 | 32.31 | 84.32 | 89321.01 |
| DINCH (ng/mL) | 69.35 | 2.87 | 18.45 | 0.45 | 0.78 | 1.23 | 3.42 | 431.56 |
| PAEs (ng/mL) | 78.25 | 412.43 | 1987.65 | 16.34 | 98.45 | 187.62 | 363.98 | 91140.32 |
| Equol (ng/mL) | 57.43 | 78.65 | 48.32 | 1.35 | 2.41 | 8.35 | 21.64 | 325.24 |
| PFOA (ng/mL) | 99.45 | 12.56 | 16.97 | 2.15 | 16.87 | 35.67 | 55.35 | 120.36 |
| PFOS (ng/mL) | 95.65 | 5.64 | 9.34 | 0.43 | 1.87 | 5.32 | 19.24 | 75.90 |
| PFDeA (ng/mL) | 47.23 | 0.65 | 1.32 | 0.12 | 0.17 | 0.21 | 0.45 | 4.35 |
| PFHxS (ng/mL) | 85.42 | 1.45 | 4.35 | 0.13 | 0.78 | 1.65 | 2.34 | 35.65 |
| PFOSA (ng/mL) | 53.21 | 0.23 | 0.56 | 0.11 | 0.15 | 0.19 | 0.20 | 3.50 |
| PFNA (ng/mL) | 43.28 | 0.32 | 0.65 | 0.07 | 0.32 | 0.64 | 0.98 | 7.87 |
| PFUA (ng/mL) | 64.34 | 0.12 | 0.32 | 0.07 | 0.07 | 0.15 | 0.27 | 6.80 |
| PFASs (ng/mL) | 76.35 | 8.65 | 5.12 | 0.43 | 26.78 | 39.34 | 47.45 | 126.89 |
| Creatinine (Cr, mg/dL) | 99.65 | 117.45 | 90.23 | 6.43 | 68.34 | 124.54 | 187.32 | 635.53 |

DEP: Diethyl phthalate; DiBP: Di–isobutyl phthalate; DnBP: Di–n–butyl phthalate; BBzP: Butylbenzyl phthalate; DEHP: Di–(2–ethyl–hexyl) phthalate; DnOP: Di-octyl phthalate; DiNP: Di-iso-nonyl phthalate; DiDP: Di-iso-decylphthalate; DEHTP: Di-2-ethylhexyl terephthalate; DINCH: Di-iso-nonyl-cyclohexane-1,2-dicarboxylate; PFOA: perfluorooctanoic acid; PFOS: perfluorooctane sulfonic acid; PFDeA: perfluorodecanoic acid; PFHxS: perfluorohexane sulfonic acid; PFOSA: 2-(N-methylperfluoroctanesulfonamido) acetic acid; PFNA: perfluorononanoic acid; PFUA: perfluoroundecanoic acid.
