# Supplementary material for: Association between endocrine disrupting chemicals and female infertility: a study based on NHANES database
Source: Front Public Health. 2025 Jun 30;13:1608861. doi: 10.3389/fpubh.2025.1608861 (PMC12257772; doi:10.3389/fpubh.2025.1608861)
Supplement: Supplementary file 2 [file Table_2.doc]

Table S2. Results of the sensitivity analysis by excluding participants whose EDCs concentrations were above the 99th percentile.

|  | Model 1 (OR) | Model 2 (OR) | Model 3 (OR) |
| --- | --- | --- | --- |
| DEP (ng/mL) | **1.11** | 1.18 | 1.16 |
| DiBP (ng/mL) | 0,85 | 0.73 | 0.74 |
| DnBP (ng/mL) | **1.80** | **1.81** | **2.06** |
| BBzP (ng/mL) | 0.92 | 0.78 | 1.23 |
| DEHP (ng/mL) | **1.58** | 1.44 | **1.35** |
| DnOP (ng/mL) | 1.36 | 1.04 | 1.36 |
| DiNP (ng/mL) | **1.35** | **1.28** | **1.59** |
| DiDP (ng/mL) | 1.53 | 1.52 | 1.71 |
| DEHTP (ng/mL) | **1.46** | **1.22** | **1.41** |
| DINCH (ng/mL) | 1.14 | 1.35 | 1.22 |
| PAEs (ng/mL) | 1.39 | 1.33 | **1.41** |
| Equol (ng/mL) | 1.48 | 1.40 | **1.36** |
| PFOA (ng/mL) | **1.12** | 1.14 | **1.31** |
| PFOS (ng/mL) | 1.53. | 1.32 | 1.17 |
| PFDeA (ng/mL) | 1.31 | 1.42 | 1.51 |
| PFHxS (ng/mL) | 1.29 | 1.74 | 1.81 |
| PFOSA (ng/mL) | 1.29 | 1.31 | 1.09 |
| PFNA (ng/mL) | 1.19 | 1.65 | 1.46 |
| PFUA (ng/mL) | **0.65** | 0.78 | **1.53** |
| PFASs (ng/mL) | 2.01 | 1.87 | 2.31 |
|  |  |  |  |

The statistically significant indices were marked in bold (P<0.05).
