# Supplementary material for: Association between endocrine disrupting chemicals and female infertility: a study based on NHANES database
Source: Front Public Health. 2025 Jun 30;13:1608861. doi: 10.3389/fpubh.2025.1608861 (PMC12257772; doi:10.3389/fpubh.2025.1608861)
Supplement: Supplementary file 3 [file Table_3.doc]

Table S3. Results of the sensitivity analysis by randomly reassigned PFAS to the lowest measurable concentration.

|  | Model 1 (OR) | Model 2 (OR) | Model 3 (OR) |
| --- | --- | --- | --- |
| DEP (ng/mL) | **1.11** | 1.19 | 1.16 |
| DiBP (ng/mL) | 0.86 | 0.71 | 0.73 |
| DnBP (ng/mL) | **1.82** | **1.82** | **2.08** |
| BBzP (ng/mL) | 0.93 | 0.79 | 1.23 |
| DEHP (ng/mL) | **1.60** | 1.45 | **1.34** |
| DnOP (ng/mL) | 1.34 | 1.02 | 1.35 |
| DiNP (ng/mL) | **1.36** | **1.31** | **1.61** |
| DiDP (ng/mL) | 1.54 | 1.53 | 1.74 |
| DEHTP (ng/mL) | **1.43** | **1.20** | **1.43** |
| DINCH (ng/mL) | 1.15 | 1.35 | 1.23 |
| PAEs (ng/mL) | 1.42 | 1.34 | **1.42** |
| Equol (ng/mL) | 1.51 | 1.42 | **1.41** |
| PFOA (ng/mL) | **1.13** | 1.12 | **1.33** |
| PFOS (ng/mL) | 1.54 | 1.33 | 1.20 |
| PFDeA (ng/mL) | 1.34 | 1.43 | 1.53 |
| PFHxS (ng/mL) | 1.31 | 1.74 | 1.82 |
| PFOSA (ng/mL) | 1.32 | 1.32 | 1.12 |
| PFNA (ng/mL) | 1.20 | 1.64 | 1.43 |
| PFUA (ng/mL) | **0.66** | 0.76 | **1.55** |
| PFASs (ng/mL) | **2.04** | 1.88 | 2.33 |
|  |  |  |  |

The statistically significant indices were marked in bold (P<0.05).
